# Complete subserosal embedding of the appendix-cecal complex presenting as a non-visualized appendix during pediatric laparoscopic appendectomy: a case report

**DOI:** 10.3389/fsurg.2026.1914887

**Published:** 2026-09-03

**Authors:** Guanghua Zhang, Ming Sun, Tao You, Hongqiang Bian, Jun Yang

**Affiliations:** 1 Department of General Surgery, Wuhan Children’s Hospital, Tongji Medical College, Huazhong University of Science and Technology, Wuhan, Hubei, China; 2 Department of Ophthalmology, Wuhan Children’s Hospital, Tongji Medical College, Huazhong University of Science and Technology, Wuhan, Hubei, China

**Keywords:** acute appendicitis, anatomical variation, intraoperative decision-making, missing appendix, pediatric laparoscopy, subserosal appendix

## Abstract

**Background:**

Failure to identify the appendix during laparoscopic appendectomy is uncommon but challenging. Complete subserosal embedding of the appendix-cecal complex is an exceptionally rare variant that may obscure all conventional laparoscopic landmarks. We describe this anomaly in a pediatric patient and describe a practical exploration strategy derived from this case.

**Case presentation:**

A 7-year-old boy presented with acute right lower quadrant pain suggestive of appendicitis. Computed tomography showed appendiceal dilatation with periappendiceal inflammation. During laparoscopic appendectomy, the appendix could not be identified despite tracing the teniae coli and complete cecal mobilization. A targeted serosal incision at the teniae convergence revealed a completely embedded appendix-cecal complex. Subserosal dissection and appendectomy were completed successfully (operative time 45 min, blood loss 2 mL). Histopathology confirmed acute suppurative appendicitis. The patient recovered uneventfully with 6-month asymptomatic follow-up.

**Conclusion:**

Complete subserosal embedding of the appendix-cecal complex is a rare variant presenting as a non-visualized appendix. Systematic exploration and awareness of unusual anatomical variants are essential. Targeted serosal incision may facilitate safe laparoscopic management and avoid unnecessary conversion to open surgery.

## 1 Introduction

Acute appendicitis ranks among the most frequent pediatric surgical emergencies and constitutes a top indication for urgent abdominal surgery in children (1). Laparoscopic appendectomy (LA) has become the primary surgical choice in most pediatric surgical centers, attributed to its advantages including milder postoperative pain, faster postoperative recovery and panoramic intra-abdominal visualization (2). Conventionally, surgeons locate the appendix by tracking three taeniae coli to their convergence point, which serves as a reliable anatomical marker for the appendiceal root (3).

A wide spectrum of congenital anatomical variations of the vermiform appendix has been described, including retrocecal, pelvic, subhepatic, ascending colonic, intramural, and intracecal locations (4–6). Although these positional variants may complicate clinical presentation or surgical exploration, most retain at least part of the appendiceal contour or recognizable anatomical landmarks that facilitate intraoperative localization. Among all reported variants, complete subserosal embedding of the appendix remains exceptionally rare (7, 8). The majority of previously reported subserosal and intramural appendiceal malformations retain partial recognizable surface markers, such as visible appendiceal base, residual mesoappendix, subtle serosal bulging or identifiable vascular pedicles, allowing successful localization during surgical exploration (9–11).

In contrast, full subserosal embedding of the entire appendix-cecal unit creates unique intraoperative difficulties (8). Under this malformation, the appendix, mesoappendix and appendiceal vascular pedicle are fully covered by intact cecal serosa, erasing all routine laparoscopic anatomical landmarks. Even with clear clinical and radiological evidence confirming acute appendicitis, surgeons may face the predicament of a “missing appendix” (11). This intraoperative dilemma may trigger diagnostic confusion, prolonged operation duration, accidental cecal wall injury, or premature conversion to open laparotomy (2). Notably, this rare malformation cannot be accurately predicted by preoperative imaging modalities, and definitive diagnosis relies entirely on intraoperative exploration findings (11).

To the best of our knowledge, no dedicated case report has specifically focused on complete subserosal embedding of the appendix-cecal complex in the pediatric population (7, 12). No publication has specifically summarized a practical exploration strategy for a non-visualized appendix during laparoscopic appendectomy (9, 13). Herein, we report a pediatric case of acute suppurative appendicitis complicated by complete subserosal embedding of the appendix-cecal complex and describe a practical stepwise exploration strategy, derived from our intraoperative experience, that may assist surgeons encountering a non-visualized appendix during laparoscopic appendectomy.

This case report adheres to the CARE Guidelines.

## 2 Case description

### 2.1 Patient information and history

A previously healthy 7-year-old boy was admitted to the emergency department with a 6-hour history of persistent dull right lower quadrant (RLQ) pain aggravated by physical activity. He denied fever, vomiting, diarrhea, constipation, and dysuria. There was no history of abdominal surgery, abdominal trauma or inflammatory bowel disease.

Physical examination showed stable vital signs: body temperature 36.5 °C, heart rate 86 beats per minute, blood pressure 110/70 mmHg. Abdominal examination revealed localized tenderness and mild rebound tenderness at McBurney's point, without muscular guarding or palpable abdominal mass. Hepatosplenomegaly was not detected. Bowel sounds were normal, and genitourinary examination was unremarkable.

### 2.2 Laboratory findings

Laboratory tests revealed a white blood cell count of 11.5 × 10⁹/L with 78% neutrophils, a C-reactive protein level of 11.8 mg/L and a procalcitonin level of 0.14 ng/mL. These findings were indicative of an acute inflammatory process. Serum electrolytes, liver and renal function tests, and routine urinalysis were all within normal ranges.

### 2.3 Imaging findings

**Figure 1:**
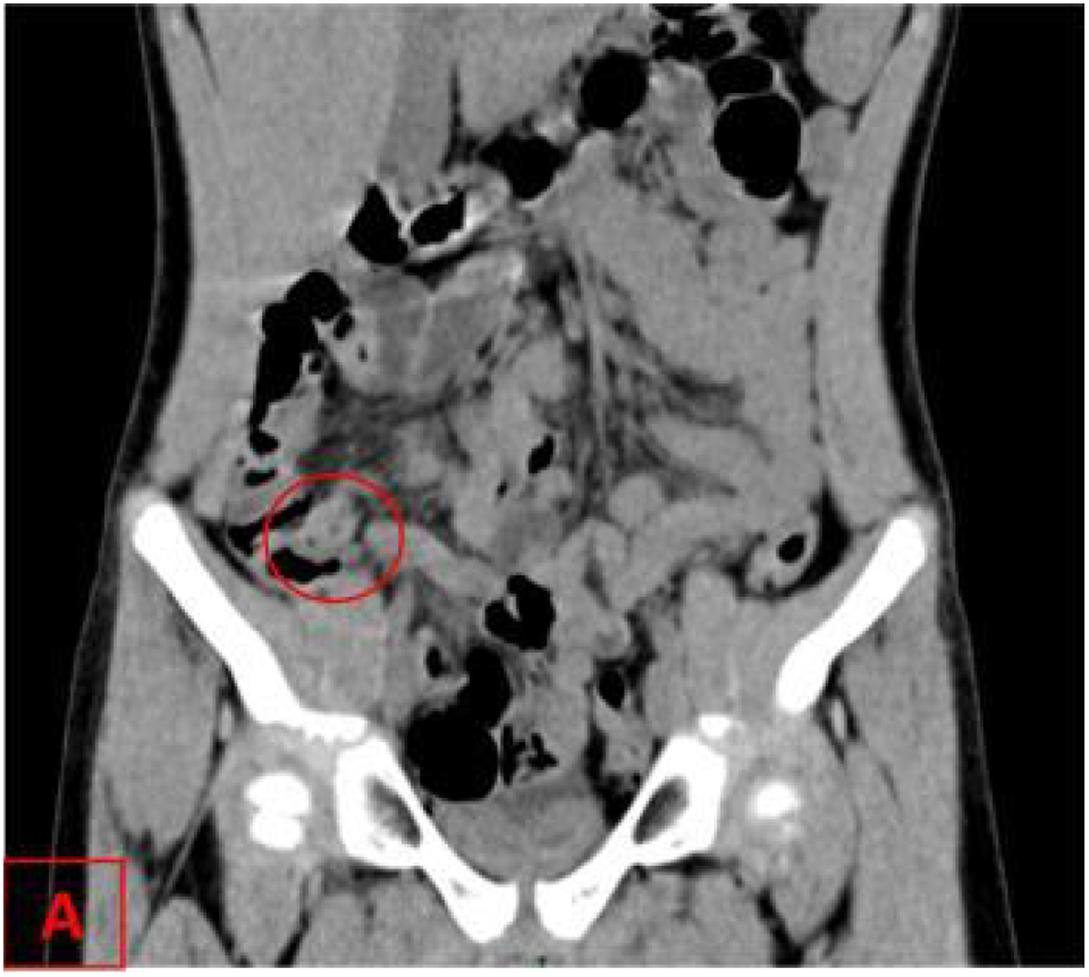
Preoperative contrast-enhanced computed tomography images. **(A)** Axial CT image demonstrating mild appendiceal dilatation (approximately 7 mm in diameter) with surrounding fat stranding (red circle), mild ileocecal wall thickening, and a small amount of pelvic free fluid, consistent with acute appendicitis.

Preoperative abdominal ultrasonography failed to visualize the appendix and demonstrated only nonspecific inflammatory changes around the cecum, without fecalith, free intraperitoneal fluid, or abscess formation.At our regional tertiary pediatric center, contrast-enhanced computed tomography (CT) is selectively performed rather than routinely used when ultrasonography is inconclusive or when additional anatomical information is required for clinical decision-making. Given the nondiagnostic ultrasonographic findings despite persistent clinical suspicion of acute appendicitis, a low-dose contrast-enhanced computed tomography (CT) scan was subsequently performed following informed consent from the patient's legal guardians. CT demonstrated a mildly dilated appendix with a terminal diameter of approximately 7 mm, accompanied by periappendiceal fat stranding and mild thickening of the ileocecal wall. A small amount of pelvic free fluid was also detected. Although typical imaging manifestations of acute appendicitis were clearly presented, the complete subserosal embedding of the appendix-cecal complex could not be identified on preoperative CT images (Figure 1).

### 2.4 Preoperative preparation and surgical setup

The patient received routine preoperative management for emergency surgery, including intravenous fluid resuscitation and prophylactic antibiotic therapy with a single dose of cefuroxime sodium at 40 mg/kg. Written informed consent for surgery and case publication was obtained from the patient's legal guardians.

The patient was placed in the supine position with Trendelenburg tilt and slight left lateral rotation. A standard three-port laparoscopic technique was adopted: a 5-mm umbilical port for the laparoscope, and two additional 5-mm working ports placed at the suprapubic region and McBurney's point respectively. Pneumoperitoneum was maintained at 8–10 mmHg throughout the operation.

### 2.5 Intraoperative findings and surgical management

#### 2.5.1 Initial laparoscopic survey

A thorough exploration of the entire peritoneal cavity was conducted to rule out other etiologies of RLQ pain, including intussusception, Meckel's diverticulitis, Crohn's disease, cecal diverticulitis, and other causes of localized ileocecal inflammation. The ileocecal region appeared normal without adhesion or abnormal fluid collection.

#### 2.5.2 Tracing the teniae coli

**Figure 2:**
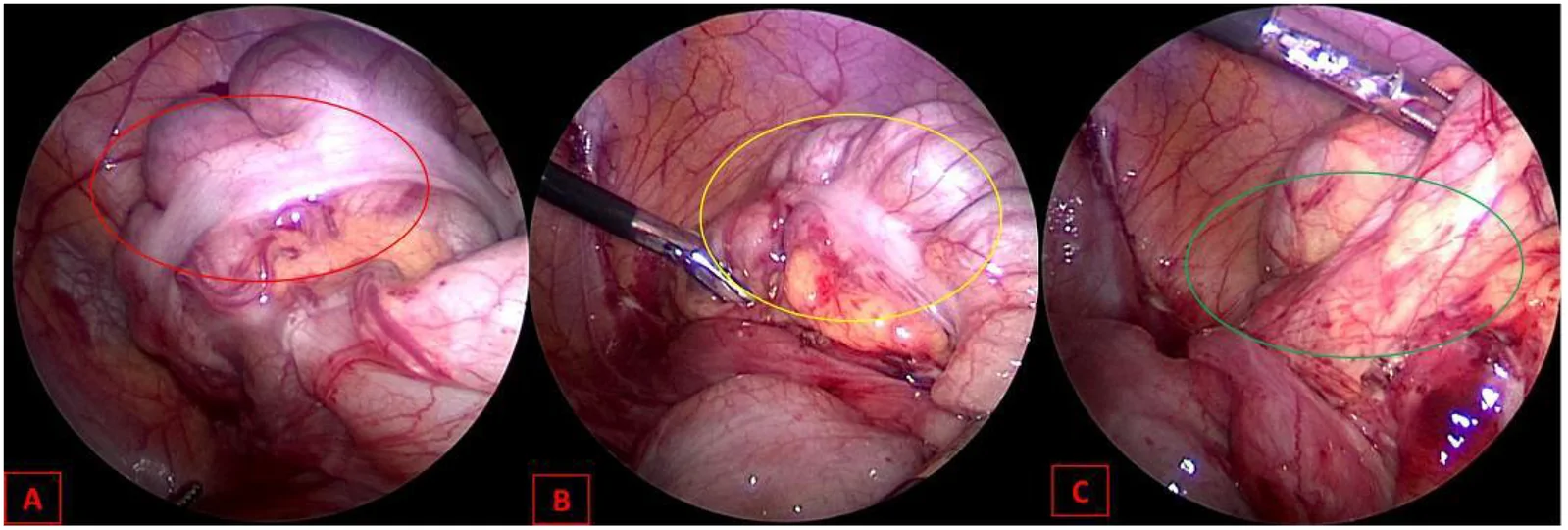
Initial laparoscopic exploration. **(A–C)** The three teniae coli (red, yellow, and green circles) are traced to their point of convergence, corresponding to the expected location of the appendiceal base. No appendiceal structure, appendiceal orifice, depression, or serosal protrusion is identified at this site.

The three teniae coli were traced until their convergence, the standard anatomical site of the appendiceal base. No appendiceal orifice, dimple or bulging mass was identified at this position (Figure 2).

#### 2.5.3 Full cecal mobilization

**Figure 3:**
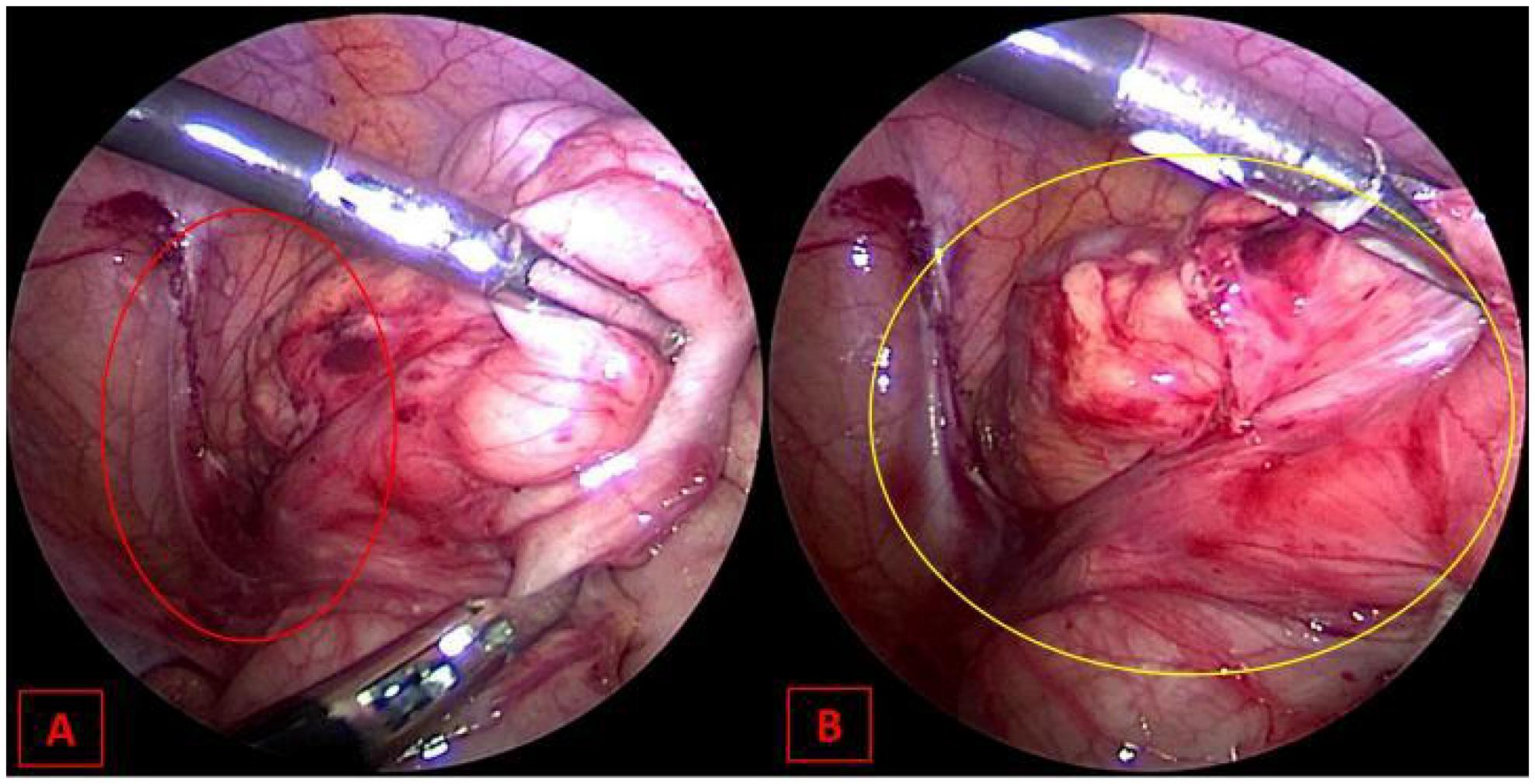
Findings after complete cecal mobilization. **(A)** Medial reflection of the cecum exposes the retrocecal space (RS) (red circle), where no appendiceal structure is identified. **(B)** The mobilized cecum demonstrates a smooth and intact serosal surface (yellow circle) without visible appendiceal landmarks.

The lateral peritoneal attachments of the cecum were divided using a low-power ultrasonic scalpel (Ethicon). The cecum was reflected medially to explore the retrocecal space, and no appendiceal tissue was found (Figure 3).

#### 2.5.4 Differential diagnosis exclusion

Further exploration revealed no retrocecal appendix, inflammatory phlegmon, or other identifiable cause of a non-visualized appendix. The ileocecal region remained free of significant adhesions or anatomical distortion.

#### 2.5.5 Targeted serosal incision and subserosal dissection

**Figure 4:**
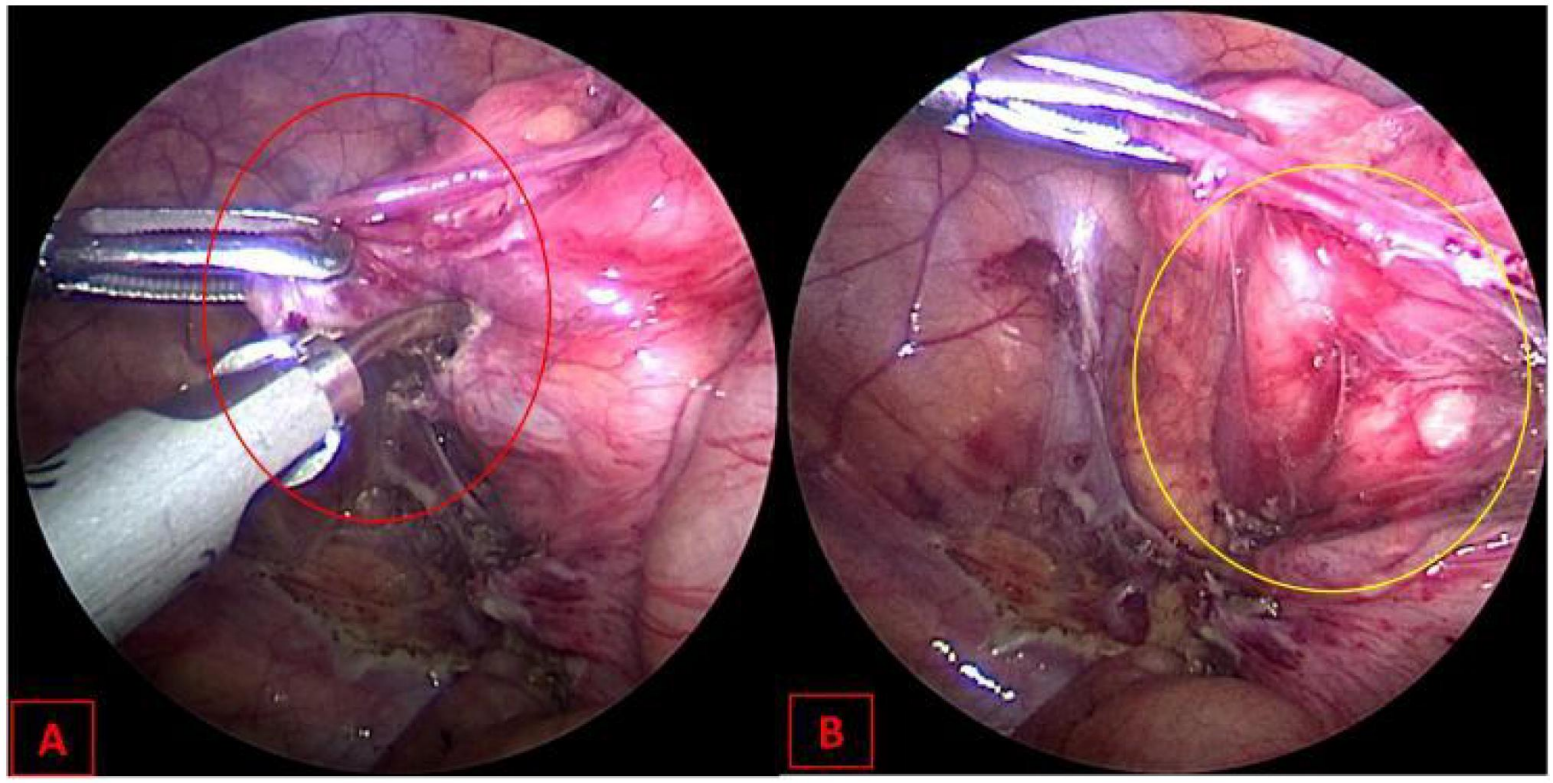
Targeted serosal incision. **(A)** A longitudinal serosal incision is created near the convergence of the teniae coli (red circle). **(B)** Following serotomy, the concealed appendiceal complex becomes visible beneath the cecal serosa (yellow circle).

Given the strong clinical suspicion of acute appendicitis, radiological evidence of localized ileocecal inflammation, complete absence of visible appendiceal landmarks despite full cecal mobilization, and failure to identify any alternative pathology, an occult appendiceal location was strongly suspected. Therefore, a targeted serosal incision was made at the convergence of the teniae coli. Following confirmation of the subserosal plane, the anatomical point corresponding to the appendiceal base (Figure 4).

**Figure 5:**
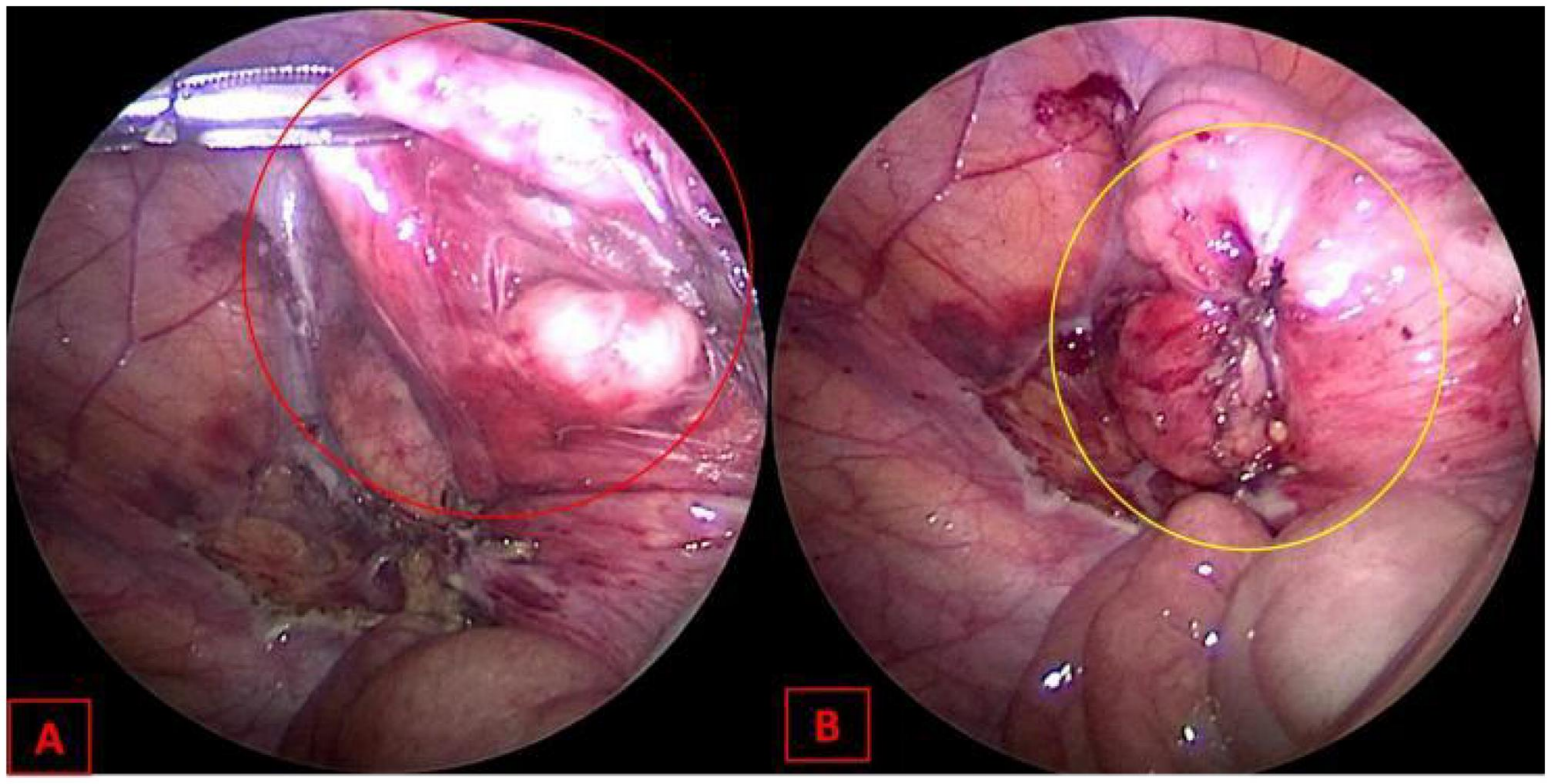
Subserosal dissection and repair. **(A)** Complete exposure of the embedded appendiceal complex, including the appendix, mesoappendix, and appendicular artery (red circle). **(B)** Closure of the serosal defect with interrupted absorbable sutures following appendectomy, restoring the integrity of the cecal wall (yellow circle).

A 1-cm longitudinal serosal incision was created at this location. Following confirmation of the subserosal plane by gentle elevation of the serosal edges, careful blunt dissection was performed using a Maryland dissector and ultrasonic scalpel while preserving the underlying cecal muscularis propria. This maneuver revealed a fibrous sheath enclosing the entire appendix (6.0 × 0.7 cm), mesoappendix, and appendicular artery (Figure 5A). The appendix was then mobilized through subserosal dissection using a technique analogous to retrograde subserosal appendicectomy, adapted for pediatric anatomy (13).

#### 2.5.6 Appendectomy and closure

The appendiceal base was ligated with 2-0 non-absorbable suture (Ethicon), and the appendix was transected approximately 0.5 cm distal to the ligation site. The appendiceal stump was inverted with a 2-0 non-absorbable purse-string suture (Ethicon). The appendicular artery was ligated and divided. The serosal defect was closed with interrupted 5-0 absorbable sutures (Ethicon) to restore the anatomical integrity of the cecal wall (Figure 5B).

### 2.6 Timeline

(Table 1).

**Table 1:** Time point clinical event.

Time Point	Clinical Event
Day 1	Onset of right lower quadrant pain and presentation to the emergency department with localized tenderness at McBurney's point
Day 1	Laboratory tests showed elevated inflammatory markers; abdominal ultrasonography failed to visualize the appendix
Day 1	Low-dose contrast-enhanced CT demonstrated a mildly dilated appendix with localized pericecal inflammation, while the congenital subserosal embedding anomaly remained undetected
Day 1	Laparoscopic exploration failed to identify the appendix; targeted serosal incision immediately revealed a completely subserosal appendix
Day 1	Subserosal dissection and laparoscopic appendectomy were successfully completed (operative time: 45 min; estimated blood loss: 2 mL)
Postoperative Day 3	Uneventful recovery; oral feeding resumed, laboratory and ultrasonographic findings normalized, and the patient was discharged
6-month Follow-up	The patient remained asymptomatic without postoperative complications or evidence of recurrence

### 2.7 Pathological findings

Gross examination of the specimen showed an appendix measuring 6.0 cm × 0.7 cm with congested and edematous serosa. Histopathological examination confirmed acute suppurative appendicitis, characterized by transmural neutrophil infiltration, mucosal ulceration and intraluminal purulent exudate. Inflammatory changes were observed in periappendiceal adipose tissue. No gangrene, perforation or neoplastic lesion was identified. Fibrous hyperplasia was noted in the mesoappendix, and the structure of the appendicular artery remained intact.

### 2.8 Postoperative follow-up and outcome

At the 6-month follow-up, the patient had normal appetite and bowel function, with normal physical growth and development for his age. Physical examination and abdominal ultrasound were unremarkable. The patient fully returned to daily activities and sports without any restrictions.

## 3 Discussion

### 3.1 Embryological considerations and anatomical significance

Complete subserosal embedding of the appendix-cecal complex may represent a congenital developmental variant. The absence of previous abdominal surgery, chronic inflammation, or other acquired anatomical abnormalities in this patient supports a congenital origin. Although the precise embryological mechanism remains uncertain, possible explanations include abnormal fusion of serosal layers during cecal development, incomplete evagination of the appendiceal primordium, or abnormal incorporation of the developing appendix into the cecal wall (5, 14). Regardless of the underlying mechanism, the resulting anatomical configuration is characterized by complete concealment of the appendix, mesoappendix, and appendicular vascular pedicle beneath an intact layer of cecal serosa (7, 8).

The clinical significance of unusual appendiceal anatomy extends beyond its rarity. Previous reports have shown that uncommon appendiceal locations, such as an ascending colonic appendix, may result in atypical clinical manifestations, misleading radiological findings, and increased diagnostic uncertainty. Nevertheless, these variants generally preserve identifiable appendiceal structures or surface landmarks that allow eventual localization during surgery. In contrast, the present case demonstrated complete subserosal embedding of the entire appendix-cecal complex, with complete concealment of the appendix, mesoappendix, and appendicular artery beneath an intact cecal serosa. Consequently, all conventional laparoscopic landmarks were absent, creating a true “non-visualized appendix” and posing a unique intraoperative challenge. In routine laparoscopic appendectomy, identification of the appendix relies heavily on visual localization through the convergence of the three teniae coli (3). When these landmarks fail to reveal the appendiceal base, the surgeon may encounter a true “missing appendix” scenario, substantially increasing diagnostic uncertainty and operative difficulty (11). Importantly, although preoperative CT can reliably establish the diagnosis of acute appendicitis based on inflammatory findings, it appears incapable of predicting this unusual anatomical variant, consistent with prior radiological descriptions of atypical appendiceal malformations (11, 15).

### 3.2 A distinct “landmark-negative” variant

As summarized in Table 2, previously reported congenital appendiceal anatomical variants—including subserosal, intramural, intracecal, ascending colonic, and ectopic appendices—generally retained at least partial anatomical clues, such as a visible appendiceal base, residual mesoappendix, subtle serosal bulging, or identifiable vascular structures, thereby facilitating intraoperative localization (6, 7, 10). A systematic review further demonstrated that nearly all reported subserosal appendices retained residual landmarks, whereas completely concealed appendix–cecal complexes without any surface markers remain exceptionally rare (7).

**Table 2:** Reported rare congenital appendiceal anatomical variants and their intraoperative characteristics.

Study	Anatomical variant	Key intraoperative finding	Recognizable appendiceal landmark	Clinical significance
Sebastian et al., 2007 (9)	Subserosal appendix (stripping)	Appendix embedded beneath cecal serosa	Partially preserved	Difficult appendectomy because of subserosal location
Abramson, 1983 (10)	Intracecal appendix	Appendix located within the cecal wall	Preserved	Rare congenital anomaly causing diagnostic confusion
Cañizares Quisiguiña et al., 2021 (7)	Complete subserosal appendix	Incidental neuroendocrine tumor in a completely subserosal appendix	Partially preserved	Extremely rare anatomical variant discovered incidentally
Munasinghe et al., 2022 (6)	Ascending colonic appendix	Appendix originated from the ascending colon	Preserved	Extremely unusual congenital positional anomaly
Teferi et al., 2024 (8)	Subhepatic, subserosal, retroperitoneal appendix	Complex ectopic appendix identified intraoperatively	Preserved	Challenging exploration because of combined positional anomalies
Present case	Completely embedded subserosal appendix	Entire appendix, mesoappendix, and appendicular artery concealed beneath intact cecal serosa	Absent (landmark-negative)	Required targeted serosal incision for identification and safe appendectomy

By contrast, the present case demonstrated complete concealment of the appendix, mesoappendix, and appendicular artery beneath an intact cecal serosa, leaving no recognizable laparoscopic landmark despite systematic tracing of the three teniae coli and complete mobilization of the cecum. From a practical surgical perspective, the defining feature of this anomaly is therefore not simply its subserosal location, but the complete absence of recognizable anatomical landmarks. To emphasize this distinctive intraoperative characteristic rather than to establish a new anatomical classification, we use the descriptive term “landmark-negative appendix”. To the best of our knowledge, this terminology has not previously been reported and is introduced solely to facilitate communication of this unique intraoperative scenario.

Accordingly, the principal challenge lies not in appendectomy itself, but in recognizing the concealed appendix when conventional localization strategies fail. An important differential diagnosis in this setting is congenital appendiceal agenesis, another rare cause of a non-visualized appendix. However, appendiceal agenesis should remain a diagnosis of exclusion and should only be considered after meticulous laparoscopic exploration has failed to identify the appendix in all potential anatomical locations. In the present case, persistent inflammatory changes confined to the ileocecal region, together with the absence of an alternative pathological process, strongly suggested that the appendix was present but concealed rather than congenitally absent. This suspicion was subsequently confirmed by targeted serosal incision, which revealed complete subserosal embedding of the appendix-cecal complex.

Recognition of this rare anatomical configuration may therefore help surgeons avoid premature diagnosis of appendiceal agenesis, unnecessary bowel resection, or excessive surgical dissection when the appendix cannot be identified during laparoscopic exploration. As summarized in Table 2, the present case appears to be unique among the reported variants in demonstrating complete loss of recognizable appendiceal landmarks, representing one of the most completely concealed appendiceal anatomical variants reported to date (8, 9).

### 3.3 Clinical implications and suggested exploration strategy

This case suggests the potential value of a structured stepwise exploration strategy when the appendix cannot be visualized during laparoscopic surgery (16). Failure to identify the appendix may lead to prolonged blind dissection, inadvertent cecal injury, or unnecessary conversion to open surgery, which remains a well-documented intraoperative complication in appendectomy cohorts (2). However, immediate serosal incision should not be regarded as a routine maneuver, and standardized exclusion steps must be completed before targeted serotomy (16).

In the present case, targeted serosal incision was considered only after completion of systematic exploration, including tracing the teniae coli, full mobilization of the cecum, inspection of the retrocecal space, and exclusion of other common explanations for a non-visualized appendix. This stepwise exploration strategy is conceptually consistent with practical approaches reported in recent case series addressing intraoperative “missing appendix” presentations (16). In the present case, this experience-based strategy facilitated safe identification of the concealed appendiceal complex while minimizing unnecessary tissue injury.

**Figure 6:**
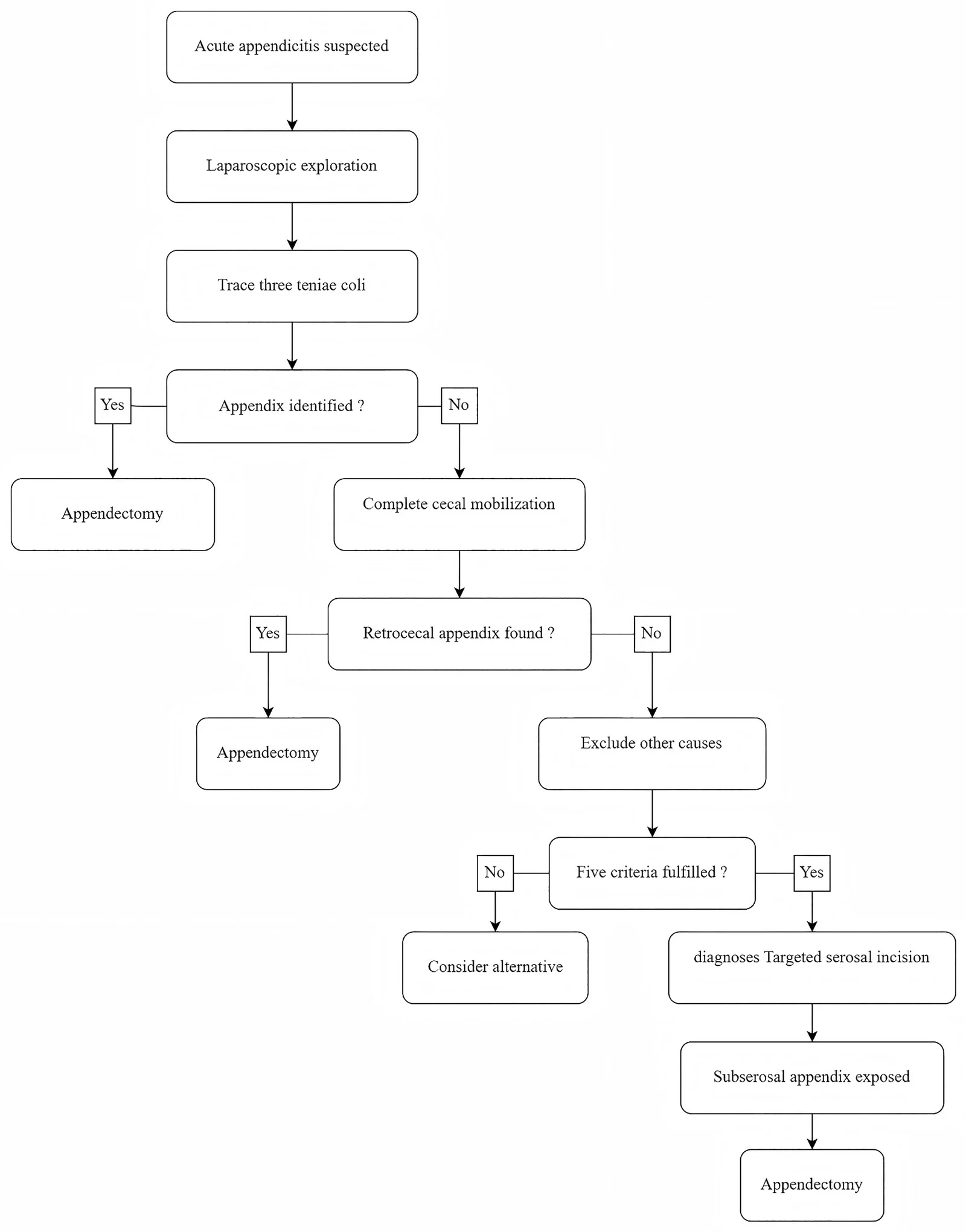
Suggested intraoperative exploration strategy for a non-visualized appendix during laparoscopic appendectomy. Decision tree summarizing an experience-based exploration strategy derived from the present case. It is intended as a practical educational reference and should not be interpreted as a validated management algorithm. Following confirmation of acute appendicitis, systematic exploration includes tracing the teniae coli, complete mobilization of the cecum, inspection of the retrocecal space, and exclusion of common causes of a non-visualized appendix. When the appendix remains unidentified despite these measures, a carefully targeted serosal incision may facilitate exposure of a completely embedded appendix-cecal complex and allow safe laparoscopic appendectomy.

Several technical principles deserve emphasis, consistent with published subserosal appendectomy techniques (9, 13, 15). First, the serosal incision should be small and precisely positioned near the convergence of the teniae coli. Second, blunt dissection should be prioritized whenever possible to preserve the underlying muscularis propria. Third, excessive electrocautery should be avoided to reduce thermal injury to the cecal wall. Finally, meticulous closure of the serosal defect is essential to restore anatomical integrity. Although derived from a single case, these practical considerations may provide useful guidance for surgeons confronted with a similar intraoperative dilemma. A summary of the suggested stepwise exploration strategy is presented in Figure 6.

### 3.4 Limitations

This report has several limitations. First, it describes a single patient, preventing any estimation of the true incidence of this rare anatomical variant; large meta-analyses of appendiceal morphometrics have excluded fully buried landmark-negative variants due to limited case volume (3). Second, the exploration strategy summarized in this report is based solely on individual clinical experience and has not been validated in larger patient cohorts, unlike consensus guidelines for standard appendicitis management (2). Therefore, it should be regarded as a practical reference rather than a standardized management protocol. Its broader applicability should be interpreted with caution until additional cases become available.Third, because extremely rare anatomical anomalies are more likely to be reported when successfully recognized and treated, publication bias is unavoidable, a recognized limitation across all single-case surgical reports (5). Additional case accumulation and multi-institutional collaboration will be required to further characterize this condition and evaluate optimal management approaches (7, 12).

## 4 Conclusion

Complete subserosal embedding of the appendix-cecal complex is an exceptionally rare anatomical variant that may present intraoperatively as a true “missing appendix” through obscuration of all conventional laparoscopic landmarks. Although preoperative imaging can establish the diagnosis of acute appendicitis, this anomaly may remain unrecognized until surgical exploration.

This case highlights the importance of maintaining awareness of rare anatomical variants when the appendix cannot be identified despite convincing clinical and radiological evidence of appendicitis. Thorough stepwise exploration, complete cecal mobilization, careful exclusion of more common causes of a non-visualized appendix, and judicious use of targeted serosal incision may facilitate safe laparoscopic identification and resection of a concealed appendix while avoiding unnecessary conversion to open surgery. Further case accumulation is needed to better characterize this rare condition and validate the practical applicability of this exploration strategy.

## Data Availability

The original contributions presented in the study are included in the article/Supplementary Material, further inquiries can be directed to the corresponding author.
